# A New Diagnostic Resource for *Ceratitis capitata* Strain Identification Based on QTL Mapping

**DOI:** 10.1534/g3.117.300169

**Published:** 2017-09-09

**Authors:** Sheina B. Sim, Raul Ruiz-Arce, Norman B. Barr, Scott M. Geib

**Affiliations:** * USDA-ARS Daniel K. Inouye U.S. Pacific Basin Agricultural Research Center, Tropical Crop and Commodity Protection Research Unit, Hilo, Hawaii 96720; † Center for Plant Health Science and Technology, Mission Laboratory, USDA-APHIS, Edinburg, Texas 78541

**Keywords:** linkage mapping, genome-wide genotyping, QTL, medfly, sterile insect technique, diagnostics

## Abstract

The Mediterranean fruit fly *Ceratitis capitata* (Wiedemann) is a destructive agricultural pest and the subject of exclusion efforts in many countries. Suppression and eradication of invasive populations to prevent its establishment is facilitated by the release of sterile males using the sterile insect technique (SIT). In SIT release areas, it is critical to accurately discriminate between released sterile males and wild individuals to detect extremely rare invasive individuals in areas inundated with millions of sterile male flies. Current methods for discrimination exist but are not always definitive, and a more reliable method is necessary. To address this, we developed a genotyping assay that can be used to discriminate between sterile males from the SIT strain and wild individuals. This was achieved by identifying single nucleotide polymorphisms (SNPs) linked to the maintained traits that facilitate male-only releases, white pupae (*wp*) and temperature-sensitive lethal (*tsl*), via QTL mapping. This resulted in the identification of one SNP that was in near-perfect linkage disequilibrium between genotype at this locus and the pupal color phenotype. Medfly from many SIT colonies and wild individuals from across its geographic range were genotyped for this locus, and results show its consistency in identifying SIT flies. In addition, linkage and QTL mapping of *wp* and *tsl* have larger impacts as they can serve as foundational tools to identify the genetic basis of traits that facilitate the separation of males from female flies, which can be used to develop SIT programs in related species.

The Mediterranean fruit fly (medfly), *Ceratitis capitata*, is an economically important agricultural pest that has the potential to inflict costly damage on a broad range of fruit and vegetable crops around the world and affect the multibillion-dollar U.S. specialty crop industry through the prohibition of fruit and vegetable exports from medfly established areas (Enkerlin 2005). One component of control programs used to prevent its establishment and contribute to its eradication in newly invaded agriculturally rich regions is the sterile insect technique (SIT). This technique operates on the premise that by inundating small invasive populations with sterile males, reproduction in the wild population will be suppressed and the population will be reduced and eventually eradicated (Knipling 1955). This process requires a mass-reared population in which sterility can be induced and subsequent sterile individuals can be released in large quantities. A classic example of this methodology was in the eradication of the New World screwworm from the United States in the mid-twentieth century (Wyss 2000; Krafsur 1998; Myers *et al.* 1998; Krafsur *et al.* 1987), and most recently, the release of SIT flies to control a new introduction of *C. capitata* in the Dominican Republic on March 2015, resulting in its swift eradication in the majority of the affected areas within 10 months (Gil 2017). Currently, SIT is employed to maintain the continental United States free from tephritid fruit fly pests such the medfly and Mexican fruit fly *Anastrepha ludens* (Franz 2005; Klassen 2005; Klassen and Curtis 2005; Hendrichs *et al.* 1995; USDA-APHIS 2010). The technique is most effective if a genetic sexing strain (GSS) is available for the target species, as a GSS facilitates the cost-effective and rapid separation of female and male flies before sterilization, resulting in male-only releases. Male-only releases are preferred when using SIT because sterile females sting host fruits in an attempt to oviposit (Hendrichs *et al.* 1995; Andrewartha and Birch 1960; Rossler 1979; Whitten 1969; Zepeda-Cisneros *et al.* 2014), causing unnecessary damage and potential infection of phytopathogenic bacteria and fruit rot fungi to crops. In addition, sterile females lower the efficacy of sterile males in successfully mating with wild females through mating competition (Hendrichs *et al.* 1995; Rendon *et al.* 2004; Zepeda-Cisneros *et al.* 2014).

In medfly, the Vienna strains (*e.g.*, Vienna-7, Vienna-8, and Vienna-8 D53-) are GSSs used for mass-release in SIT applications, particularly in Southern California and Central America. In Vienna strains, there are two sexually dimorphic and tightly linked traits: white pupae (*wp*) and temperature sensitive lethal (*tsl)*. Female Vienna medfly have a mutant white pupae phenotype, in contrast to males, which have a wild-type, brown pupae phenotype. The white pupae phenotype was first found in the fifth filial generation of an inbred colony that originated from a cross between an irradiated male and a wild-type female (Rossler 1979). In addition to being homozygous for *wp*, female Vienna medfly are also homozygous for a temperature-sensitive lethal mutation, a mutation originally induced into colony flies through the feeding of ethyl methanesulphonate (Busch-Petersen 1990). Homozygosity for *tsl* causes females to die at temperatures > 33°, which is in contrast to males who are heterozygous at this locus and thus survive at elevated temperatures (Kerremans and Franz 1995). When eggs produced by the Vienna lines undergo a high-temperature treatment (*e.g.*, incubation in an aerated water bath at 34° for 24 hr), the hatching larvae are essentially all male, and any surviving females can be identified at the pupal stage and excluded based on their white pupal color. Recombinants (brown pupae and temperature-sensitive individuals or white pupae nontemperature-sensitive individuals) are rare due to the tight linkage between the genes that govern these two traits and lowered recombination in males (Morgan 1914; Rossler 1982). Due to the artificial conditions under which these mutations and phenotypes were created, the likelihood of the mutations causing white pupae and temperature-sensitivity occurring in wild populations is very low.

Although these two traits are autosomal recessive and should not be displayed in a sex-specific manner, sex linkage is maintained through a reciprocal translocation between the autosome carrying the two causative genes (*wp* and *tsl*, which are on chromosome 5) and the Y sex chromosome in males (Figure 1) (Franz 2005). Vienna-7 and Vienna-8 flies differ in the location of the translocation breakpoint, and Vienna-8 flies contain a chromosomal inversion that places the gene causing *tsl* in closer physical proximity to the gene causing *wp*; this is in contrast to Vienna-8 D53- which lacks this inversion. The reciprocal translocation in Vienna flies, coupled with low recombination in males, preserves all males in a wild-type heterozygous state, and all females as homozygous recessive (Franz *et al.* 1994).

**Figure 1:**
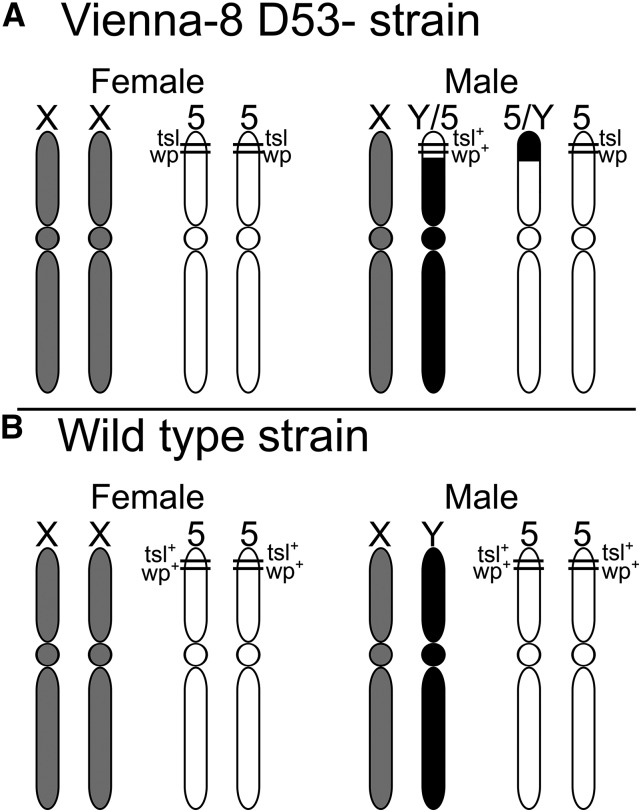
(A) Schematic of the sex chromosomes and the fifth chromosome containing the *wp* and *tsl* gene for female and male Vienna-8 GSS (genetic sexing strain) and HiMed *C. capitata*. The location of the *white pupae* and *temperature-sensitive lethal* gene has been crudely mapped to the tip of the fifth chromosome, and a translocation between the Y-chromosome and the fifth chromosome harbors a wild-type allele (*wp*^+^) in males and is heterozygous at the locus, in contrast to females who are homozygous for the *wp* and *tsl* mutations. (B) Drawing of the sex chromosomes and the fifth chromosome containing the genes for female and male wild-type *C. capitata white pupae* and wild-type *temperature-sensitive lethal*; wild-type individuals lack the *wp* and *tsl* variants. This figure is adapted from Franz (2005).

Mass-produced sterilized males are released into areas at high risk of medfly invasion, and this practice is one of several components of an integrated pest management (IPM) program. An additional component of IPM is population monitoring, which is achieved by hanging a high density of yellow sticky card insect traps in high-risk areas (Klassen 2005). Due to the volume of traps that require attention, it is not practical to check traps daily, and the insects that are caught are subject to predation and weathering.

In areas with an active SIT program, such as Southern California, several hundred million SIT flies are released each week on a continuous basis, and intensive trapping in these areas results in a recapture rate of 0.02–0.07%, depending on trapping density (USDA-APHIS 2014). This amounts to 1–2 million flies that are recaptured yearly that must be differentiated from wild medfly captures, which may only be present at very low population levels. This is, in part, achieved through the marking of radiation-sterilized pupae with a powdered fluorescent dye, a marking method developed by Norris (1957) and subsequently modified for use in SIT programs by Steiner (1965) and Schroeder *et al.* (1972) (Enkerlin *et al.* 1996). Although the dye is eventually removed from the insect body through grooming, contact of the cephalic ptilinum sac with the dye upon adult emergence results in integration of the dye into the head of the fly when the sac is retrieved back into the head (Enkerlin *et al.* 1996). Because of the localization of the dye in the head, fluorescent marking can be unreliable upon the recapture of partial (headless) or weathered flies.

Another method used by regulatory agencies to identify trapped material involves the dissection of gonadal tissue. This requires the examination of captured flies by a trained specialist to determine if its condition is consistent with that of irradiated flies (Guillén Aguilar 1983). Unfortunately, this is a process that can become costly and time consuming if the number of captured flies are numerous. Though these two methods are useful in screening flies, additional techniques are needed to diagnose flies that are in poor condition and lack the diagnostic marking with dye.

In an effort to address this need, molecular methods have been developed. However, these various methods have all relied upon mitochondrial haplotypes that are associated with Vienna flies (San Andres *et al.* 2007; Barr 2009; Parubrub *et al.* 2015). For example, an assay based on a protocol reported by Barr (2009) requires sequencing of a 584 bp region of the mitochondria, which includes subunits 5 and 4 of the NADH gene. Examination of various Vienna strain flies from SIT colonies at multiple mass-rearing facilities showed that all reared Vienna flies exclusively have one of two haplotypes for the mitochondrial N5N4 locus; the M006 haplotype is found in the Vienna-8 strain reared by the Moscamed facility in El Piño, Guatemala, and both the M006 and M007 haplotypes are found in the Vienna-8 D53- strain reared by the California Department of Food and Agriculture (CDFA) in Waimanalo, HI. This method can diagnose a captured fly as a wild fly (*i.e.*, not a Vienna genetic sexing strain) if the unknown sample has any haplotype apart from M006 and M007 at the N5N4 locus. However, the low but detectable presence of the M006 and M007 haplotypes in the wild (R. Ruiz-Arce, unpublished data) prohibits the unequivocal designation of a captured fly as an Vienna strain fly. This limitation in diagnosis is also a problem for the other mitochondrial methods because the diagnostic genotypes reported are not private to Vienna strain colonies.

The purpose of this project was to design a diagnostic assay that can be used in addition to the N5N4 locus to discriminate between wild and Vienna strain flies. To achieve this goal, linkage and QTL analysis was performed to identify a locus that is tightly linked to traits maintained in Vienna strain colonies. This resulted in the development of a single nucleotide polymorphism (SNP) genotyping assay that discriminates between individuals based on the white pupae phenotype, a phenotype with artificial origins, which increases its likelihood of being exclusive to the Vienna strain.

In addition to working toward the development of a diagnostic assay to support the discrimination of wild flies from released individuals, the results of this study will be used as a foundation that can direct future genetic research on the medfly and related species. The identification of the loci closely linked to *wp* can be used as a starting point for the future identification the genes governing *wp* and *tsl*, the causative mutations underlying the mutations, and targets for recreating these phenotypes in new species to rapidly create genetic sexing strains in other important insect pests. In addition, this map can be applied to a recently published medfly genome (Papanicolaou *et al.* 2016) to further improve this assembly and place scaffold regions and genes into a chromosomal context.

## Materials and Methods

### Test crosses and fly husbandry

**Figure 2:**
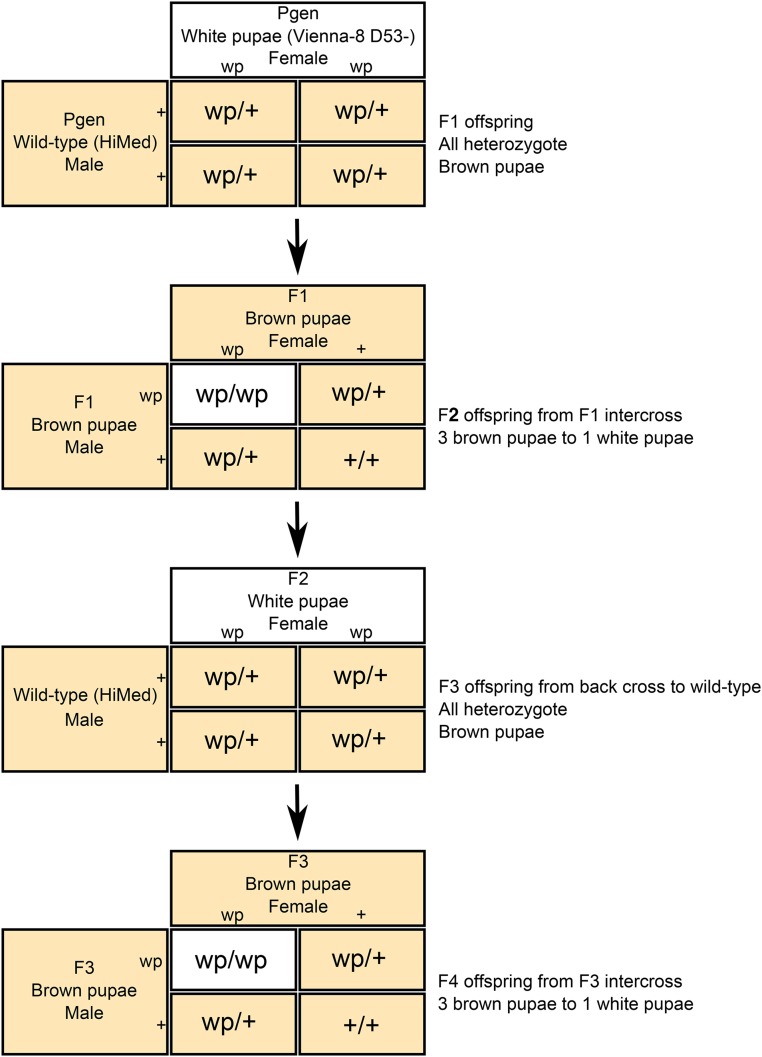
Virgin adult females from the *C. capitata* genetic sexing strain (Vienna-8 D53-) were mated in isolation with males from the wild-type laboratory colony (HiMed). The *white pupae* trait (wp) is autosomal recessive, so resulting F1 progeny will all have a wild-type brown pupal color phenotype. In F2 progeny from isolated intercrossing between F1 full-siblings, the pupal color phenotype will segregate at a 3:1 ratio of wild-type brown pupae to white pupae. White pupae F2 females were back-crossed to wild-type laboratory colony males. This increases the proportion of the wild-type alleles genome in subsequent offspring. Like the F1 progeny, the F3 progeny will all have a wild-type brown pupal color phenotype and full-sibs will be intercrossed to produce an F4 mapping population comprised of female and male wild-type brown pupae and white pupae individuals. Colors of the text boxes denote pupal color of individuals, white boxes represent white pupae individuals, and yellow boxes represent brown pupae individuals.

The GSS flies used to perform the genetic test crosses were Vienna-8 D53- strain flies obtained from the CDFA mass-rearing facility in Waimanalo, HI. Wild-type individuals were from a colony called “HiMed” that are reared and maintained at the United States Department of Agriculture-Agricultural Research Service (USDA-ARS) facility in Hilo, HI. Individual flies from the Vienna-8 D53- and HiMed lines were used as parents to create three sibships representing mapping populations. The crossing scheme employed (Figure 2), produced recombinant offspring with nonsex-linked variation in pupal color (white or wild-type brown) in an inbred, mostly wild-type background. To generate the mapping population to map *white pupae*, virgin Parental (P) generation Vienna-8 D53- white pupae females were mated with virgin wild-type HiMed males in isolated crosses. The resulting F1 population were wild-type and heterozygous for *wp*, and were intercrossed to recover the white pupae phenotype. The resulting white pupae F2 females were backcrossed to the wild-type HiMed colony males in isolated matings. The resulting F3s, which were heterozygous at the *wp* locus, were intercrossed to recover the white phenotype. This method of generating a mapping population decoupled the white pupae phenotype from sex due to the exclusion of the reciprocal translocated autosome and Y chromosome through usage of wild-type colony males, which have a wild-type chromosomal arrangement. This crossing scheme was replicated and produced many sibships, with the three most productive sibships used for genotyping experiments.

The adult flies used in this experiment were reared at the USDA-ARS under conditions similar to those described by Vargas (1989), but with minor amendments. Adult *C. capitata* were given a diet of a 3:1 ratio of white sugar and yeast hydrolysate and reared in 350 ml screen-covered cups. Each cup contained isolated male and female pairs, or male only or female only cohorts, from single sibships. Larvae were given a diet consisting of 26.3% wheat mill feed, 12.2% granulated white sugar, 3.4% torula yeast, 2.3% citric acid, 0.2% nipagen (preservative), 0.2% sodium benzoate (preservative), and 59.5% water. Individuals of all life stages were held in a room maintained at 25° with 65% humidity.

### DNA extraction and genotype by sequencing library production

Individual F4 flies from the three most productive lines, along with their parents (from the F3 generation), F2 source matings, and the original parental (P generation) individuals were used for genotype by sequencing (GBS) library preparation (Elshire *et al.* 2011). DNA extraction was performed on individual adult flies. Whole flies were homogenized in tissue lysis buffer using a FastPrep 24 homogenizer (MP Biomedical, Santa Ana, CA) for 20 sec at 4.0 m/sec. Homogenized samples were incubated in a 55° water bath for 3 hr, followed by DNA extraction on a Kingfisher Flex 96 automated extraction instrument (Thermo Scientific, Waltham, MA), using standard protocols with a Mag-Bind Tissue DNA KF Kit (Omega, BioTek, Norcross, GA). In addition to samples derived from the crossing scheme, an additional 169 samples were included representing flies derived from major *C. capitata* mass rearing colonies and a broad geographic distribution of wild flies (Supplemental Material, Table S1). The quantity and quality of the extracted DNA samples were determined using the High Sensitivity Genomic DNA Analysis Kit on a Fragment Analyzer (Advanced Analytical, Ankeny, IA).

DNA extracted from all individuals was assessed for quantity and integrity, and high-quality samples were sent for library preparation at the Institute for Diversity GBS facility at Cornell University, Ithaca, NY. Library preparation was performed by ligating barcodes unique within a plate of samples to genomic DNA digested with the restriction enzyme *Ape*KI. Illumina HiSeq 2500 was used to generate single-end 102 bp reads with in-line barcodes included in raw sequences. Raw sequences from the mapping population are available on NCBI, associated with BioProject PRJNA341700 and BioSamples SAMN05725882 to SAMN05725907 and SAMN05727687 to SAMN05727802. Raw sequences from samples from the wild populations and Vienna fly colonies are also available and associated with BioProject PRJNA341535 and BioSamples to SAMN05725834 to SAMN05725997 and SAMN05726002 to SAMN05726006. The BioSample accessions associated with the sequences from each individual are listed in Table S1.

### SNP discovery

Raw GBS reads for each sample were individually aligned to a *C. capitata* whole-genome assembly (Papanicolaou *et al.* 2016), made available through the 5000 Insect Genome Project (I5K) (https://i5k.nal.usda.gov/, NCBI WGS accession AOHK00000000.1, BioProject accession PRJNA201381). GBS tags were aligned to the reference scaffold assembly using the Burrows–Wheeler Alignment Tool (Li and Durbin 2009). SNP discovery and genotyping was performed using the *TASSEL* pipeline V3.0 (Glaubitz *et al.* 2014). The plugins *FastqToTagCountPlugin*, *MergeMultipleTagCountPlugin*, *TagCountToFastqPlugin*, *SAMConverterPlugin*, *FastqToTBTPlugin*, *MergeTagsByTaxaFilesPlugin*, *TagsToSNPByAlignmentPlugin*, *MergeDuplicateSNPsPlugin*, and *GBSHapMapFiltersPlugin* were used to demultiplex GBS sequences, align fragments, identify SNPs, assign genotypes for every locus for each individual, and filter genotypes based on read depth and a minimum minor allele frequency. Default parameters were used, and SNP genotype filtering was based on a minimum minor allele frequency of 0.05, no linkage disequilibrium with at least one neighboring SNP, low taxon coverage, and high heterozygosity. Further filtering was performed using *VCFtools* v0.1.13 (Danecek *et al.* 2011), and SNP genotypes were included in the analysis on the condition that there was ≤ 50% missing data after excluding individuals with low sequence coverage. Initial assessment of differentiation between the two colonies used in the mapping populations, HiMed (*N* = 6 females and 6 males) and Vienna-8 D53- (*N* = 12 males), was performed by estimating Weir and Cockerham’s Fst using *VCFtools* v0.1.13 (Danecek *et al.* 2011) for all identified SNP loci and a Manhattan plot of Fst values was generated using the R package *qqman* (Turner 2014).

### Population genetic analysis of SNP genotypes from GBS library

After excluding individuals and SNP loci that contained > 20% missing data, a population genetic analysis on 143 individuals from several sources was performed using their genotypes for 77,844 SNP loci. These 143 individuals were from several mass-rearing Vienna fly colonies, the wild-type colony HiMed, wild flies collected from regions where there are no current Vienna fly strain releases (the Azores, Brazil, Hawaii, Honduras, Morocco, Mozambique, Panama, Reunion Island, and Senegal), and flies collected from regions with active SIT, where Vienna fly strain are released (Australia, California, Guatemala, and Spain). A discriminant analysis of principle components (DAPC), which clusters individuals in a data set to maximize the variation between clusters and minimizes variation within clusters, was performed using the R package *adegenet* v.2.0.2 (Jombart 2008; Jombart and Ahmed 2011). The number of genetic clusters that describe these 143 individuals was identified using the function *find.clusters* and assignment of each individual to a cluster was performed by the *dapc* function using 20 principle components and six discriminant functions.

### Linkage and QTL mapping

The SNP genotypes obtained from the *TASSEL* pipeline were used to estimate a linkage map featuring chromosome-scale linkage groups. This analysis was performed using the recombination mapping algorithms of *LEP-Map2* (Rastas *et al.* 2013, 2015), which simultaneously processes large amounts of SNP genotype data for several mapping populations. It was written specifically for use with genome-wide SNP data and organisms with achiasmatic meiosis, such as insects in the orders Lepidoptera and Diptera. Custom python scripts were used to modify existing SNP data in *.hmp* format into a *LEP-Map2*-compatible format. Using *Lep-MAP2*, genome-wide SNP genotypes from all parents and progeny from three mapping populations were filtered for Mendelian errors using custom python scripts and the *Filtering* module. Loci were then assigned to linkage groups using the *SeparateChromosomes* module. A minimum of 10 loci was required for a linkage group to be reported. Ungrouped SNP loci were included using the *JoinSingles* module, and the *OrderMarkers* module was used to order the markers within each linkage group defined by *SeparateChromosomes*. Due to the lowered and nearly absent recombination in male Diptera (Morgan 1914; Rossler 1982), the initial recombination rate for males was set to 1.0e−9 in the *OrderMarkers* module, while for females it was set to 0.05.

The outputs of *LEP-Map2* were modified with Python utilities to create files compatible with the *qtl* package in *R* (Broman 2003; Broman *et al.* 2003) and visualized using the *LinkageMapView* package, also in *R* (Ouellette *et al.* 2017). Loci linked to the *wp* locus were identified through implementation of the single-locus binary QTL model, a model that is most appropriate for the *wp* trait and is described by Xu and Atchley (1996). Following this, a permutation test using 100,000 permutations was performed according to described methods to identify significant loci above the threshold of 1e−4 (Broman 2003; Broman *et al.* 2003). This shows the minimum logarithm of the odds (LOD) score a locus can have and still be considered significantly linked to the trait. The SNP loci displaying the strongest linkage to the *wp* phenotype as determined by a permutation test was used to design TaqMan SNP genotyping assays to differentiate between white pupae and wild-type brown pupae individuals.

Population genetic differentiation analysis was performed between Vienna strain individuals from around the world and wild flies. To do so, Weir and Cockerham’s Fst was estimated between Vienna strain individuals (*N* = 63) collected from multiple mass-rearing facilities and flies collected from the wild (*N* = 88) in the established range of the medfly. For this analysis, only SNP loci in the linkage map were included to be able to identify which regions of the genome are highly diverged between Vienna strain flies and wild flies.

### TaqMan genotyping assay design

The SNP locus displaying the strongest linkage to the *wp* phenotype as determined by a permutation test was located on scaffold NW_004523946.1 at base position 1,353,742, on the superscaffold that corresponds to chromosome 5. To confirm the validity of this analysis, a TaqMan SNP genotyping assay (Thermo Scientific) was designed around this marker. The assay, designed and identified as AHMSY8D by Thermo Scientific, was designed with the forward primer sequence 5′-GGAAAGGAAATAAGGTGGAGTAACCA-3′, reverse primer sequence 5′-GGTGTTGAAGTGTTGACTAGACATG-3′, wild-type allele reporter (VIC) sequence 5′-CAGCGCAGCACCGG-3′, and mutant allele reporter (FAM) sequence 5′-CAGCGCATCACCGG-3′. Based on SNP genotypes determined from GBS libraries for the mapping population, Vienna strain individuals, and wild individuals, the wild-type allele is the nucleotide G at the SNP locus in contrast to the nucleotide T, which is only present in Vienna strain individuals with Vienna strain females being homozygous for T and Vienna strain males being heterozygous.

### Genotype confirmation of assay on Vienna colony flies and wild-derived flies

The initial genotype confirmation tests for TaqMan custom assay AHMSY8D took place at the USDA-ARS Daniel K. Inouye US PBARC laboratory in Hilo, HI. Amplifications were performed in triplicate in volumes of 10.0 μl containing 5.0 μl SensiFAST Hi-ROX Genotyping Kit (Bioline, London, UK), 0.5 μl 20× TaqMan SNP assay, 2.0 μl template DNA, and 2.5 μl DNAse-free ${\text{H}}_{2}\text{O}.$ The qPCR thermal cycling was performed on a StepOnePlus Real-Time PCR system (Thermo Scientific) using a fast protocol under the following cycling conditions: initial pre-PCR read stage at 25.0° for 30 sec; 95.0° for 20 sec; 40 cycles of 95.0° for 3 sec and 60° for 20 sec; and a final post-PCR read stage at 25.0° for 30 sec. Data analysis on the change (Δ) in fluorescence for both reporters relative to the passive dye and assignment of genotype was performed on the StepOne Software v2.3 (Thermo Scientific). A total of 43 individuals from the Vienna-7 colony (*N* = 21) and the HiMed colony (*N* = 24) were genotyped. Additionally, three of the wild captures from Spain that clustered with mass-rearing Vienna strains in the DAPC analysis were genotyped to determine consistency between GBS and the TaqMan assay AHMSY8D in showing that genotypes observed in those flies were consistent with Vienna strain genotypes.

### Cross-platform genotype confirmation

Additional genotype confirmation tests of the TaqMan assay AHMSY8D was performed on Vienna strain individuals collected from multiple mass-rearing facilities to demonstrate that the assay is consistent among the different Vienna colonies and that the assay is compatible across different qPCR platforms. At the USDA-APHIS CPHST Mission Laboratory in Edinburg, TX, qPCR amplifications were performed in replicates in volumes of 10.0 μl containing 5.0 μl 2× Perfect Real Time Premix ExTaq (Takara Bio USA, Mountain View, CA), 0.25 μl 40× Perfect Real TimePremix ExTaq Buffer, 3.75 μl DNAse-free ${\text{H}}_{2}\text{O},$ and 1.0 μl template DNA. Thermal cycling for these reactions were performed on the CFX-96 Touch (Bio-Rad, Hercules, CA) using the same amplification conditions as what was performed by USDA-ARS described above. A total of 113 available individuals collected from several Vienna fly colonies reared in mass-rearing facilities in Austria (*N* = 4), Guatemala (*N* = 75), USA (*N* = 30), and Spain (*N* = 3) were tested using this assay.

### Data availability

Demultiplexed GBS sequences are stored and curated at NCBI under BioProject accession numbers PRJNA341700 and PRJNA341535. Supporting data including the RQTL analysis file, raw linkage map, and *.vcf* are included among the supplemental files Table and Figure legends are available in File S1.

## Results

### Genetic crosses

Genetic crosses between individual *wp* females from the Vienna-8 D53- GSS colony with individual males from the HiMed wild-type colony resulted in the segregation of *white pupae* alleles and placed the mutant white pupae and wild-type brown pupae phenotypes in a common genetic background. Three sibships were generated using this isolated-mating crossing scheme, and these sibships were comprised of white pupae and wild-type individuals. The proportion of wild-type brown pupae individuals to white pupae individuals was recorded at every generation. The parental generation was comprised of wild-type brown pupae males and white pupae females. As expected, 100% of the F1 and F3 generation exhibited the wild-type brown pupae phenotype. The F2 and F4 generation that were the progeny of F1 and F3 full-sibling intercrosses, respectively, was comprised of brown and white pupae individuals at a ratio that did not significantly deviate from 3:1 (adjusted *p*-value >0.05) (Table 1) which is the ratio expected from single-heterozygote crosses. In each generation, there was no significant bias in sex-ratio. All sibships were derived from full-sibling matings and all full-sibling pairs were derived from one isolated mating pair.

**Table 1:** Ratios of wild-type brown pupae to white pupae individuals for each of four genetic crosses (names of each cross are in ID column) in F2 and F4 sibships from the genetic crossing experiment

ID	Generation	Total	Brown	White	$\chi^{2}$	*P*-Value*^a^*
8.3	F2	25	19	5	0.22	0.64
8.3.2.1	F4	114	94	20	3.38	0.07
8.3.2.2	F4	27	18	9	1.00	0.32
8.3.2.5	F4	22	15	7	0.55	0.46

a A $\chi^{2}$ test was performed to test if the deviation of observed from expected values was significantly different and resulting *p*-values are reported.

### SNP discovery

To obtain needed sequence coverage from the samples in the GBS libraries, a total of three lanes of Illumina HiSeq were used for sequencing, and 95 individuals were sequenced on each lane. The number of raw reads, reads removed due to lack of restriction site, reads removed due to low quality, and number of reads retained is included in Table S1. A total of 77,844 SNPs were identified on 443 unique scaffolds from the *C. capitata* genome assembly. Genome-wide Fst values show 368 fixed loci (Fst = 1) and 781 additional nearly fixed loci (Fst > 0.95) when comparing individuals from the wild-type HiMed colony to those from the Vienna-8 D53- GSS colony across the genome. The large number of fixed and nearly fixed SNPs that segregate between the wild-type colony and the Vienna fly colony demonstrates the need for performing a cross to isolate the differences that are attributed to the pupal color phenotype. For this application, a genome-wide association study would have identified many candidate loci that are differentially fixed in the two colonies. Furthermore, since Vienna strain males are heterozygous and thus contain a wild-type allele, the locus of interest is not expected to be differentially fixed between the two colonies when considering both males and females.

### Population genetics

**Figure 3:**
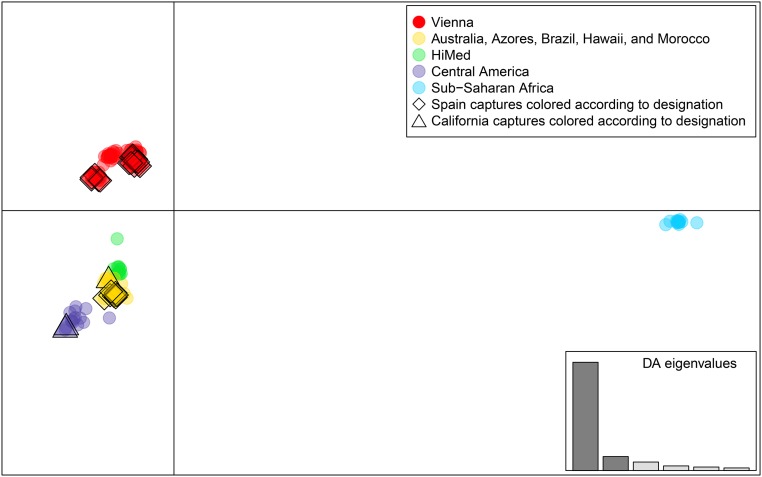
A discriminant analysis of principle components was performed on a total of 143 individuals from various sources using their genotypes for 77,844 single nucleotide polymorphism loci. All individuals have a membership probability of < 99% to one of seven clusters, Vienna strain flies were assigned to three clusters (red), and wild individuals formed four distinct clusters (yellow, green, violet, and blue). Individuals from Spain (diamonds) are denoted as some were assigned to the same cluster as Vienna strain flies (red diamonds), and some were assigned to the wild fly cluster consisting of flies originating from Australia, the Azores, Brazil, Hawaii, and Morocco (yellow diamonds). Individuals from California (triangles) are denoted as one was assigned to the wild fly cluster consisting of flies originating from Australia, the Azores, Brazil, Hawaii, and Morocco (yellow triangle), while others were assigned to the cluster containing individuals from Central America (violet traingles). The box denoting eigenvalues shows the relative variance described by each of the principle components in descending order and that the clusters can be differentiated using principle components 1 and 2 (*x*-axis and *y*-axis, respectively), which together explains 86% of the variance. The clusters roughly correspond to a geographic location, with flies collected from sub-Saharan African countries being the most distinct.

A DAPC analysis showed that the dataset, including 143 individuals from various SIT facilities that produce various Vienna strain flies, individuals from its native and established ranges in Australia, the Azores, Brazil, Hawaii, Morocco, Spain, Central America (Guatemala, Honduras, and Panama), and Africa (Mozambique, Reunion Island, Senegal, and South Africa), and three individuals collected from the southern California exclusion zone (with active SIT) (Table 2) was predicted to be from seven distinct clusters. These clusters are differentiated largely by principle components 1 and 2, which, together, explain 86% of the variance. The Vienna fly strains were divided into three clusters instead of being grouped into one (red in Figure 3). This is potentially due to the practice of colony infusion of wild material that has been adopted by the mass-rearing facilities, as the introduction of wild genetic material from different sources will result in genotypic variation that is tied to the facility rather than the strain. Individuals from the wild-type HiMed colony (green) that were used in the genetic crosses are distinct, but are most similar to individuals from Australia, the Azores, Brazil, Hawaii, Spain, and Morocco, which themselves form a distinct cluster (yellow). Individuals from Guatemala, Honduras, and Panama are distinct and form a Central American cluster (violet). Lastly, individuals from sub-Saharan Africa are also very distinct (blue). Flies caught from California (triangles) appear to be from multiple geographic sources, including both Central America and the cluster containing flies from Australia, the Azores, Brazil, Hawaii, Morocco, and Spain, with no individuals appearing to be derived from Vienna flies released for SIT. In contrast, a total of 17 of the 24 captures from Spain (red diamonds) were assigned to the Vienna fly clusters, which indicates that they are released Vienna flies, likely as part of a suppression program, that were recaptured but designated as wild through other methods.

**Table 2:** Genotyping methods used to obtain genotypes of Vienna colony flies from multiple rearing facilities

Genotyping Method	Origin of Samples	N
GBS	Mass-reared SIT colonies, wild, unknown	143
Assay AHMSY8D by USDA-ARS	Vienna-8 D53-, wild-type colony	45
Assay AHMSY8D by USDA-APHIS	Mass-reared SIT colonies	75

GBS, genotype by sequencing; SIT, sterile insect technique; USDA-ARS, United States Department of Agriculture-Agricultural Research Service; USDA-APHIS, United States Department of Agriculture-Animal and Plant Health Inspection Services.

### Linkage and QTL mapping

The three sibships in the F4 mapping population were comprised of white pupae and wild-type brown pupae individuals in a ratio that did not significantly deviate from the predicted ratio for three wild-type brown pupae to one white pupae individuals (Table 1). All sibships were derived from full-sibling matings and all full-sibling pairs were derived from one isolated mating pair. Linkage analysis placed 980 SNPs from 133 unique scaffolds onto 11 linkage groups ranging from 2.1 to 144 Mb in size and represent 71.1% of the assembled genome. Linkage groups were anchored to chromosomes based on existing polytene chromosome maps using genes found in both maps. Unplaced linkage groups were assigned letters A–D (Figure S1). There are known to be five autosomes and two sex chromosomes (X and Y) in *C. capitata* and linkage groups from this data study were anchored to three of the five chromosomes.

**Figure 4:**
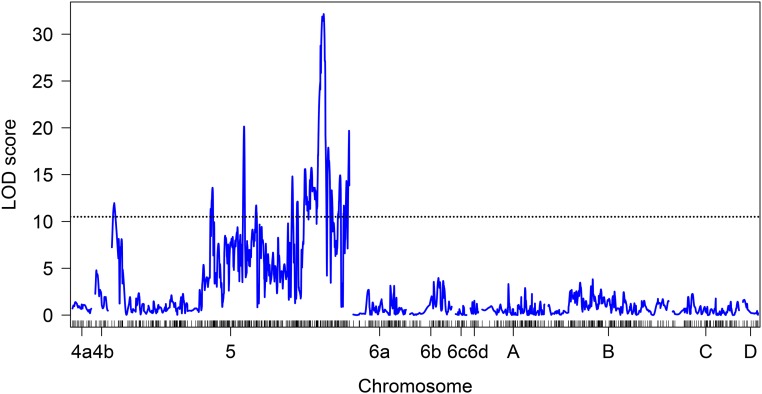
Quantitative trait loci analysis using the binary interval mapping model. Results indicate that the pupal color phenotype is tightly linked to loci on autosome 5. The dotted line represents a logarithm of the odds (LOD) threshold for significance as determined by a permutation test performed with 100,000 permutations (p < 1e−4).

Quantitative trait locus analysis on the resulting linkage map was performed using the pupal color phenotype of each individual in the mapping populations. The single locus binary model revealed tight linkage between loci on scaffold NW_004523946.1 of the *C. capitata* genome (Figure 4), which is ∼3.3 MB in length. The single locus with the largest LOD score was on this scaffold at position 1,353,742 and was attributed a LOD score of 32.2. Manual inspection of the genotypes at this locus shows that out of 114 individuals included in the QTL analysis, 109 (96%) of the genotypes were in perfect LD with the phenotype, there was 1 (< 1%) recombinant, and 4 (3.5%) genotypes were missing. The SNP locus with the highest LOD score is not located in a gene, and it is thus unlikely that the locus with the highest LOD score contains the causative mutation that confers the white pupae phenotype. However, it is likely that the gene for *white pupae* is one of the 113 genes located in the same scaffold (scaffold NW_004523946.1) as this QTL. Inspection of this scaffold for genes in the previously identified arthropod melanization pathway (van’t Hof and Saccheri 2010) revealed that none are located on the same scaffold as the SNP with the highest LOD score.

**Figure 5:**
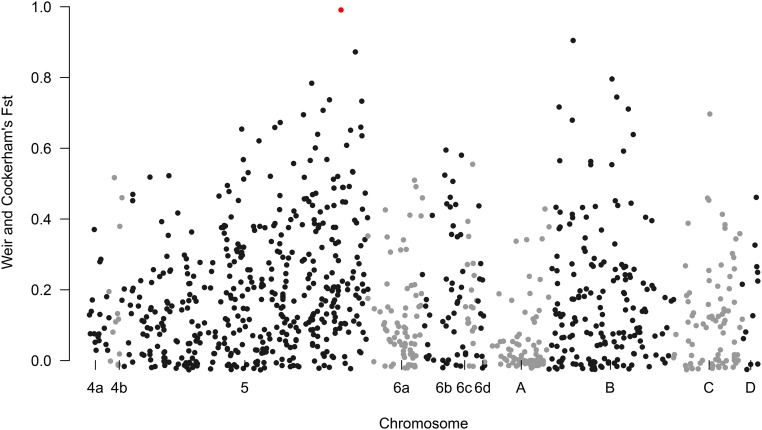
Weir and Cockerham’s Fst calculated between wild individuals and only Vienna colony females that are homozygous for *wp* using the 981 single nucleotide polymorphism (SNP) loci in the linkage map. The SNP locus identified through quantitative trait loci analysis as tightly linked to *wp* was colored red.

Calculation of Weir and Cockerham’s Fst as a measurement of differentiation between female flies from multiple Vienna colonies from around the world and wild individuals collected from the world-wide distribution of *C. capitata* shows a high level of differentiation for multiple loci. The SNP locus with the highest QTL score (the data point highlighted in red Figure 5) is identified as nearly fixed (Fst = 0.99). In our sampling of wild flies, one individual from Brazil was heterozygous at SNP locus position 1353742 on scaffold NW_004523946.1. This indicates that the allele frequency of the SNP linked to *wp* is likely ≤ 0.5% in wild *C. capitata* based on the populations sampled in this study.

### Genotype confirmation tests

**Figure 6:**
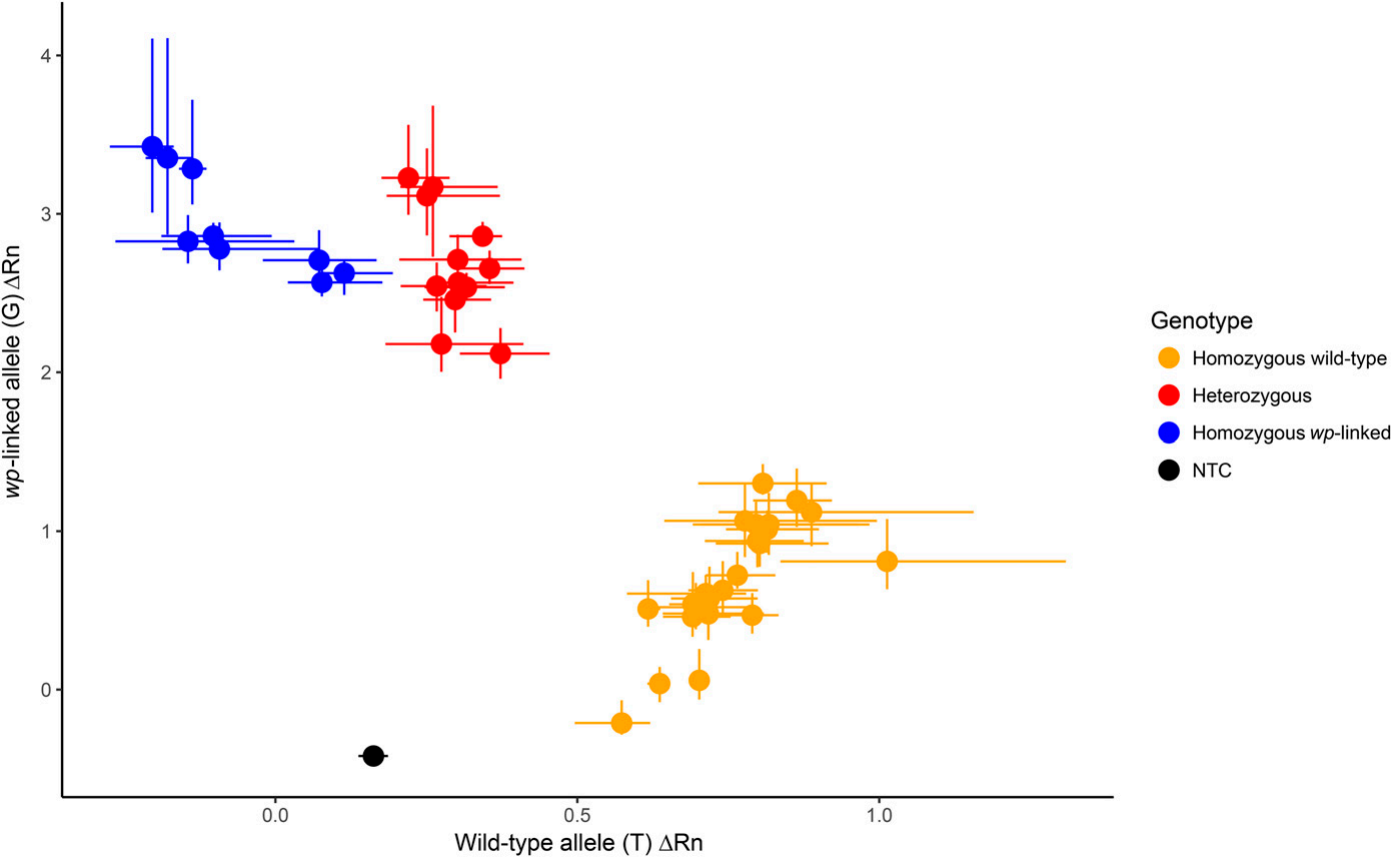
Allelic discrimination plot showing Δ Rn value for wild-type allele (T) on the *x*-axis *vs.* the Δ Rn value for the *wp*-linked allele (G) on the *y*-axis for individuals genotyped with assay AHMSY8D, where the Δ Rn value is the detected change between the initial read and the endpoint read in fluorescent signal of the reporter and the passive reference dye. Average values of three replicates are shown with minimum and maximum Δ Rn values for both alleles are shown as bars on their respective axes. Individuals that were assigned the genotype of homozygous for the wild-type allele are in orange, heterozygotes are in red, homozygous for the *wp*-linked allele are in blue, and the no template control (NTC) is in black.

Genotype confirmation tests of TaqMan assay AHMSY8D, designed to differentiate between homozygous wild-type (G/G), heterozygous (G/T), and homozygous for the *wp*-linked variant (T/T) at the *wp*-linked SNP locus, showed consistency between marker genotype and phenotype of tested individuals. A summary of the number of individuals genotyped using GBS and the developed assay can be found in Table 2. In a preliminary test of nine Vienna-8 D53- females, 12 Vienna-8 D53- males, 12 HiMed females, 12 HiMed males, and one no template control, all females from the Vienna-8 D53- GSS colony, which have the white pupae phenotype, genotyped as homozygous for the *wp*-linked allele; all males from the Vienna-8 D53- GSS colony, which have the wild-type brown pupae phenotype, genotyped as heterozygous; and all females and males from the HiMed wild-type colony, which have a wild-type brown pupae phenotype, genotyped as homozygous for the wild-type allele (Figure 6). The three wild captures from Spain, which were assigned to one of the clusters containing mass-reared Vienna strain flies based on genome-wide SNP data, showed consistency between the GBS genotype and the SNP assay genotype (Figure S2), and provides evidence that those wild-captures were Vienna strain flies released for SIT. Genotype confirmation testing of the TaqMan assay AHMSY8D on flies from several Vienna strain-rearing facilities showed that all the Vienna strain males genotyped were heterozygous and all the Vienna strain females were homozygous for the *wp*-linked allele (Table 3).

**Table 3:** Genotypes of Vienna colony flies from multiple rearing facilities

Country	City	Strain	Sex	N	Genotype
Austria	Seibersdorf	Vienna-8	Male	4	G/T
Guatemala	El Piño	Vienna-8 D53-	Male	33	G/T
Guatemala	El Piño	Vienna-8 D53-	Female	3	T/T
Guatemala	Guatemala City	Vienna-7	Male	22	G/T
Guatemala	Guatemala City	Vienna-7	Female	17	T/T
USA	Waimanalo, HI	Vienna-8 D53-	Male	30	G/T
Spain	Valencia	Vienna-8 D53-	Male	3	G/T

The nucleotide G is the wild-type allele at the locus genotyped by assay AHMSY8D, and the nucleotide T is the *wp*-linked allele.

## Discussion

The medfly is a major pest of worldwide economic importance. Their threat to high-value agriculture is so extensive that IPM programs are employed to prevent their long-term establishment in many countries worldwide. An important component of these IPM programs is the use of the SIT, which leverages the availability of GSS that are established for these species. When SIT flies are recaptured in area-wide monitoring efforts, it is necessary to be able to differentiate wild from SIT flies to prevent the initiation of unnecessary and costly quarantines when a SIT fly is mistaken for wild and also appropriately initiate quarantines when a wild fly is detected. Identification of loci linked to traits maintained in SIT released flies can be developed into a diagnostic assay, which will ensure that the appropriate management practices take place.

To address this objective, we identified SNPs that are tightly linked to a trait maintained in Vienna fly colonies that are released for SIT. These SNPs were identified for the purpose of developing a diagnostic assay that is capable of distinguishing wild flies from intentionally released SIT genetic sexing flies. This was achieved through an experimental design that generated a mapping population that was genotyped for SNPs through sequencing and analysis of a GBS library. These genotypes were used to perform a population genetic analysis that showed very strong population structure between Vienna fly colony individuals and wild individuals, and between wild individuals collected from the breadth of the geographic range of *C. capitata*. The GBS genotypes were then used to assemble scaffolds into a linkage map in addition to performing a QTL analysis. The results of the QTL analysis were used to design a diagnostic SNP assay (TaqMan assay AHMSY8D), which is commercially available for purchase, but can also be genotyped using alternative methods using the sequence information reported. This assay was tested using known Vienna strain samples to assess the consistency of the locus and demonstrate that the assay is consistent in correctly identifying Vienna fly colony individuals from various mass-rearing facilities. Additional validation studies are planned for assay AHMSY8D to measure the probability of detecting a Vienna strain profile in wild flies and the exclusiveness of the *wp*-linked allele. In addition, SNP genotypes derived from GBS of wild individuals show that the presence of the *wp*-linked allele in the wild is ≤ 0.5% when 109 individual flies from 13 countries were sampled. Although we are not able to conclude that the SNP linked to the *wp* is unique to Vienna strains, this result demonstrates the ability of this assay to be effective in differentiating wild flies from Vienna flies in all of the geographic locations sampled. It also demonstrates that the combination of this assay with mitochondrial data such as the N5N4 locus will make an effective and robust diagnostic assay.

Population genetic analysis using all 77,844 SNP loci that were determined through GBS shows two clusters of mass-rearing Vienna strain flies that are distinct, but females from both clusters are homozygous for the *wp* (T/T) allele and males are heterozygous (G/T) at the SNP assay locus. This demonstrates that the designed SNP assay can be used by all medfly diagnostic stations regardless of which Vienna-SIT strain is released. If diagnosis of particular Vienna strains is needed, then SNP profiles for additional loci would need to be generated and compared.

In addition to working toward the development of a diagnostic assay, we assembled the medfly genome into a linkage map using the mapping population used to identify the loci linked to *white pupae*. This linkage map can be used to further scaffold the assembly into chromosome-scale superscaffolds and perform comparative genomic analysis to identify synteny of orthologous genes with the model species *Drosophila melanogaster* or other tephritid species for which chromosome-scale assemblies exist (Sim and Geib 2017; Sved *et al.* 2016).

Identification of the gene causing the *white pupae* mutation in medfly is important to the development of new strategies for genetic sexing strains in the future. A limitation of our QTL analysis through genotyping with GBS sequences is that restriction site-associated SNP genotypes are unlikely to be in coding regions and a SNP locus tightly linked to the trait of interest is unlikely to be in the gene governing the trait. Differential expression analysis of RNA sequences coupled with low-coverage resequencing of white and brown pupae individuals are currently being pursued to identify the gene causing *white pupae* in medfly. Differential expression analysis of brown and white pupae individuals collecting over a time-course will identify genes or coexpression networks that match the pupal color phenotype and identify the relative time frame at which the genes are expressed (Love *et al.* 2014; Robinson *et al.* 2010; McCarthy *et al.* 2012; Langfelder and Horvath 2008, 2012; Conesa *et al.* 2006; Nueda *et al.* 2014). Low-coverage resequencing will identify all mutations in the genes or coexpression networks that are differentially expressed and the effects of these mutations can be identified (Cingolani *et al.* 2012). Once the candidate genes are identified, they can be validated using RNAi (Schroder 2003; Hughes and Kaufman 2000; Brown *et al.* 1999) or targeted mutagenesis techniques such as CRISPR-Cas9 (Friedland *et al.* 2013; Hwang *et al.* 2013; Wang *et al.* 2013; Bassett and Liu 2014). These studies are facilitated by the development of the mapping population of the project presented here, which places the white pupae trait in a common background and is thus ideal for differential expression and dense variant identification because the analysis can be controlled for variation independent of the phenotype of interest.

### Conclusions

The diagnostic tool developed based on a SNP locus tightly linked to the *wp* allele will hopefully be utilized to improve diagnostic capabilities for medfly, and help to increase the efficiency and accuracy of identification of wild medfly in regions subjected to SIT for suppression, eradication, and exclusion. By integrating this tool with existing genetic and morphological methodologies, fewer cases of inconclusive origin of recaptured flies will exist, reducing unnecessary quarantines caused by potentially recaptured Vienna SIT flies. While we expect this method to be robust, as this method is adopted and applied throughout the world to increasing numbers of geographic populations and mass-reared Vienna flies, the strength of this assay will continue to be tested and validated. This research is also the starting point to examining the causative mutations for the genetic sexing traits, and possible application of those traits to new species. In order to have an effective SIT program, a GSS that is cost-effective and facilitates the release of male-only sterile flies needs to be established for the species of interest. The identification of the *white pupae* gene in medfly can be used to identify its orthologous genes in other economic pests and the same genome editing tools used for validation can recreate this phenotype and sex linkage in other economically important species (Chen *et al.* 2015). This project provides a foundation for the development of GSS lines for important tephritid pests that are currently not amenable to male-only mass rearing for SIT.

## Supplementary Material

Supplemental material is available online at www.g3journal.org/lookup/suppl/doi:10.1534/g3.117.300169/-/DC1.
