# Supplementary figures and images for: A New Diagnostic Resource for Ceratitis capitata Strain Identification Based on QTL Mapping
Source: G3 (Bethesda). 2017 Sep 9;7(11):3637–47. doi: 10.1534/g3.117.300169 (PMC5677166; doi:10.1534/g3.117.300169)

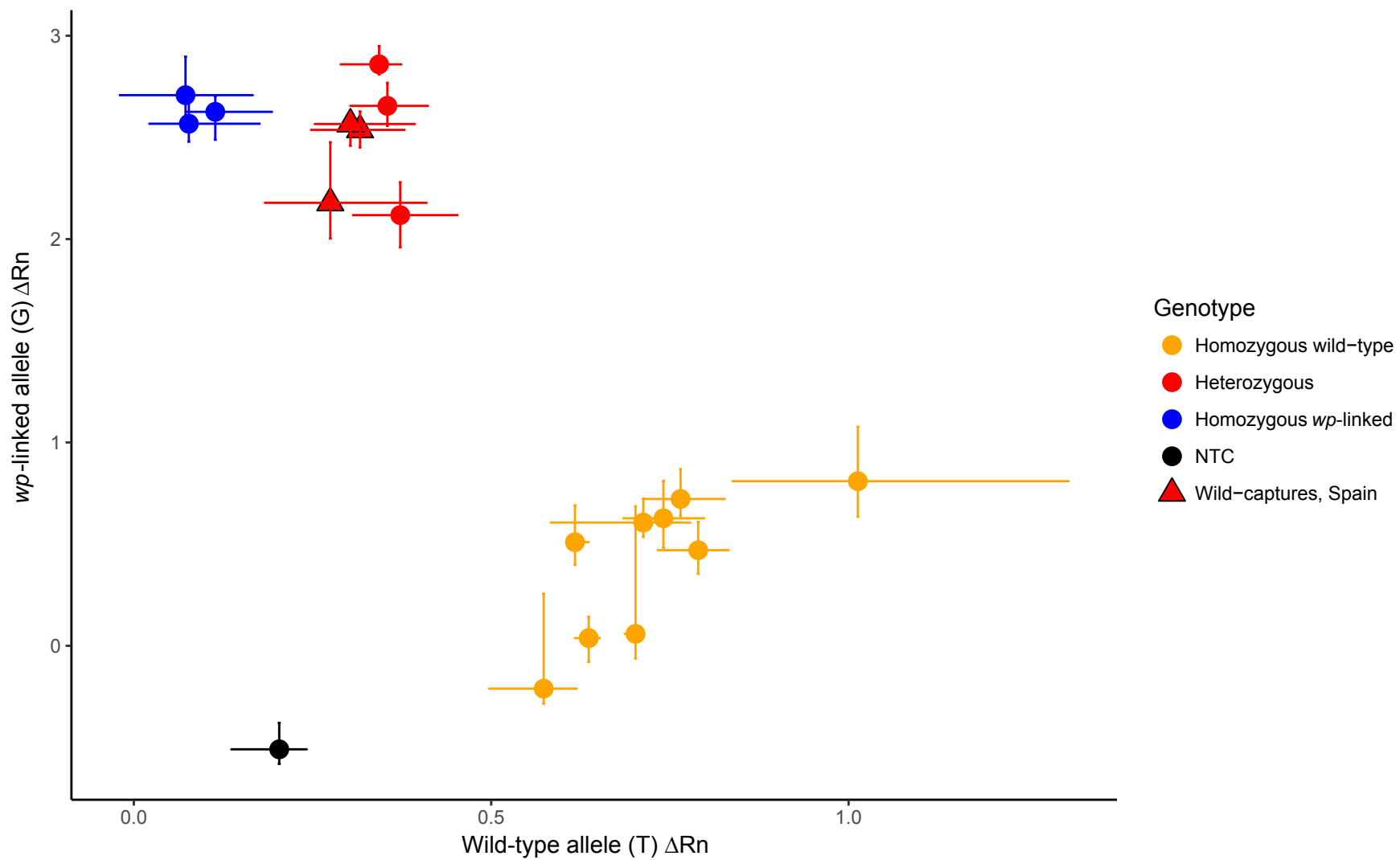

Supplement: Supplementary file 2 [file 3637FigureS2.pdf]
