# Supplementary material for: A New Diagnostic Resource for Ceratitis capitata Strain Identification Based on QTL Mapping
Source: G3 (Bethesda). 2017 Sep 9;7(11):3637–47. doi: 10.1534/g3.117.300169 (PMC5677166; doi:10.1534/g3.117.300169)
Supplement: Supplementary file 3 [file 3637TableS1.pdf]

S1 Table. Read counts other information for all individuals in the GBS library.

Individual ID	BioSample	Origin	Total	Retained	Assay AHMSY8D Genotype
20100810_001_KFZ_001_011	SAMN05725834	Brazil Sao Luis	592627	551239 (93%)	G/G
20100810_001_KFZ_001_014	SAMN05725835	Brazil Sao Luis	897386	833202 (92%)	G/G
20100810_001_MNS_009_006	SAMN05725836	Brazil Cacador Santa Catarina	3420652	3038829 (88%)	G/G
20100810_001_MNS_009_007	SAMN05725837	Brazil Cacador Santa Catarina	921848	849507 (92%)	G/G
20100810_001_MNS_009_008	SAMN05725838	Brazil Cacador Santa Catarina	1279161	1160664 (90%)	G/G
20100810_001_MNS_009_010	SAMN05725839	Brazil Cacador Santa Catarina	2002951	1855594 (92%)	G/T
2011121_001_AA001_004	SAMN05725840	Azores Terceira	2549374	2368958 (92%)	G/G
2011121_001_AA001_005	SAMN05725841	Azores Terceira	3005289	2742045 (91%)	G/G
20130628_001_E_001_009	SAMN05725842	Vienna-7 Strain Moscamed Guatemala	1727035	1609751 (93%)	G/T
20130628_001_E_001_010	SAMN05725843	Vienna-7 Strain Moscamed Guatemala	1553212	1418741 (91%)	G/T
20130628_001_E_001_011	SAMN05725844	Vienna-7 Strain Moscamed Guatemala	1235388	1161740 (94%)	G/T
20130628_001_E_001_012	SAMN05725845	Vienna-7 Strain Moscamed Guatemala	1091020	1013986 (92%)	G/T
20130628_001_F_001_013	SAMN05725846	Vienna-7 Strain Moscamed Guatemala	1697034	1578366 (93%)	T/T
20130628_001_F_001_014	SAMN05725847	Vienna-7 Strain Moscamed Guatemala	2222363	2098573 (94%)	T/T
20130628_001_F_001_015	SAMN05725848	Vienna-7 Strain Moscamed Guatemala	1275653	1206533 (94%)	T/T
20130628_001_F_001_016	SAMN05725849	Vienna-7 Strain Moscamed Guatemala	2080534	1943550 (93%)	T/T
20130628_001_I_001_017	SAMN05725850	Vienna-8 Strain South Africa SIT Africa	1667572	1524201 (91%)	G/T
20130628_001_I_001_018	SAMN05725851	Vienna-8 Strain South Africa SIT Africa	1837878	1719455 (93%)	G/T
20130628_001_I_001_019	SAMN05725852	Vienna-8 Strain South Africa SIT Africa	1521211	1412882 (92%)	G/T
20130628_001_I_001_020	SAMN05725853	Vienna-8 Strain South Africa SIT Africa	900639	844495 (93%)	G/T
20130628_001_J_001_021	SAMN05725854	Vienna-8 Strain South Africa SIT Africa	1491727	1412678 (94%)	T/T
20130628_001_J_001_022	SAMN05725855	Vienna-8 Strain South Africa SIT Africa	1144423	1079312 (94%)	T/T
20130628_001_J_001_023	SAMN05725856	Vienna-8 Strain South Africa SIT Africa	1651100	1560516 (94%)	T/T
20130628_001_J_001_024	SAMN05725857	Vienna-8 Strain South Africa SIT Africa	1954392	1823354 (93%)	T/T
20130628_001_M_001_025	SAMN05725858	Vienna-8 Strain Mix 2006 Strain. Chile	867801	806887 (92%)	G/T
20130628_001_M_001_026	SAMN05725859	Vienna-8 Strain Mix 2006 Strain. Chile	1048860	967984 (92%)	G/T
20130628_001_M_001_027	SAMN05725860	Vienna-8 Strain Mix 2006 Strain. Chile	1614546	1503544 (93%)	G/T
20130628_001_M_001_028	SAMN05725861	Vienna-8 Strain Mix 2006 Strain. Chile	1010662	915691 (90%)	G/T
20130628_001_N_001_029	SAMN05725862	Vienna-8 Strain Mix 2006 Strain. Chile	1013656	947725 (93%)	T/T
20130628_001_N_001_030	SAMN05725863	Vienna-8 Strain Mix 2006 Strain. Chile	1452468	1354634 (93%)	T/T
20130628_001_N_001_031	SAMN05725864	Vienna-8 Strain Mix 2006 Strain. Chile	1806142	1700774 (94%)	T/T
20130628_001_N_001_032	SAMN05725865	Vienna-8 Strain Mix 2006 Strain. Chile	1501554	1431086 (95%)	T/T
20130628_001_S_001_033	SAMN05725866	Vienna-7 Mix 1999 Strain. Western Australia	1721318	1623198 (94%)	G/T
20130628_001_S_001_034	SAMN05725867	Vienna-7 Mix 1999 Strain. Western Australia	2090526	1954853 (93%)	G/T
20130628_001_S_001_035	SAMN05725868	Vienna-7 Mix 1999 Strain. Western Australia	1224432	1153205 (94%)	G/T
20130628_001_S_001_036	SAMN05725869	Vienna-7 Mix 1999 Strain. Western Australia	1624361	1523318 (93%)	G/T
20130628_001_T_001_037	SAMN05725870	Vienna-7 Mix 1999 Strain. Western Australia	1075615	1004652 (93%)	T/T
20130628_001_T_001_038	SAMN05725871	Vienna-7 Mix 1999 Strain. Western Australia	779122	731599 (93%)	T/T
20130628_001_T_001_039	SAMN05725872	Vienna-7 Mix 1999 Strain. Western Australia	1060208	999308 (94%)	T/T
20130628_001_T_001_040	SAMN05725873	Vienna-7 Mix 1999 Strain. Western Australia	628746	577493 (91%)	T/T
20130628_001_Y_001_041	SAMN05725874	Vienna-7 New Strain. Seibersdorf	488817	461270 (94%)	G/T

Individual ID	BioSample	Origin	Total	Retained	Assay AHMSY8D Genotype
20130628_001_Y_001_042	SAMN05725875	Vienna-7 New Strain. Seibersdorf	667744	583615 (87%)	G/T
20130628_001_Y_001_043	SAMN05725876	Vienna-7 New Strain. Seibersdorf	1416323	1319020 (93%)	G/T
20130628_001_Y_001_044	SAMN05725877	Vienna-7 New Strain. Seibersdorf	1407113	1326614 (94%)	G/T
20130628_001_Z_001_045	SAMN05725878	Vienna-7 New Strain. Seibersdorf	875554	812640 (92%)	T/T
20130628_001_Z_001_046	SAMN05725879	Vienna-7 New Strain. Seibersdorf	1306463	1243850 (95%)	T/T
20130628_001_Z_001_047	SAMN05725880	Vienna-7 New Strain. Seibersdorf	1541109	1453674 (94%)	T/T
20130628_001_Z_001_048	SAMN05725881	Vienna-7 New Strain. Seibersdorf	2138158	2010116 (94%)	T/T
8F	SAMN05725882	CDFA	2785569	2591048 (93%)	T/T
8M	SAMN05725883	HiMed	2574976	2089626 (81%)	G/G
CDFA10	SAMN05725884	CDFA	1703931	1581903 (92%)	G/T
CDFA11	SAMN05725885	CDFA	1579076	1474251 (93%)	G/T
CDFA13	SAMN05725886	CDFA	1077977	1005370 (93%)	G/T
CDFA14	SAMN05725887	CDFA	1291375	1210231 (93%)	G/T
CDFA15	SAMN05725888	CDFA	1840198	1733829 (94%)	G/T
CDFA16	SAMN05725889	CDFA	2104798	1987399 (94%)	G/T
CDFA17	SAMN05725890	CDFA	2008803	1903728 (94%)	G/T
CDFA1	SAMN05725891	CDFA	1446839	1356710 (93%)	G/T
CDFA4	SAMN05725892	CDFA	2093586	1976602 (94%)	G/T
CDFA5	SAMN05725893	CDFA	1079025	1015913 (94%)	G/T
CDFA6	SAMN05725894	CDFA	1460525	1379351 (94%)	G/T
CDFA8	SAMN05725895	CDFA	1233232	1134970 (92%)	G/T
HIMed12	SAMN05725896	HiMed	2012420	1887206 (93%)	G/G
HIMed13	SAMN05725897	HiMed	1639148	1427295 (87%)	G/G
HIMed14	SAMN05725898	HiMed	2714191	2499696 (92%)	G/G
HIMed15	SAMN05725899	HiMed	999672	924865 (92%)	G/G
HIMed16	SAMN05725900	HiMed	1206472	1129843 (93%)	G/G
HIMed2	SAMN05725901	HiMed	3064319	2773667 (90%)	G/G
HIMed3	SAMN05725902	HiMed	1378705	1273176 (92%)	G/G
HIMed4	SAMN05725903	HiMed	1541580	1435665 (93%)	G/G
HIMed5	SAMN05725904	HiMed	1476654	1394658 (94%)	G/G
HIMed6	SAMN05725905	HiMed	1127347	1049800 (93%)	G/G
HIMed7	SAMN05725906	HiMed	1482483	1399220 (94%)	G/G
HIMed8	SAMN05725907	HiMed	1874859	1757179 (93%)	G/G
S01_14	SAMN05725908	Guatemala San Juan Atoenango Finca Capetillo[a]	1948783	1850428 (94%)	G/G
S01_16a	SAMN05725909	USA CA San Jose Santa Clara County[a]	1175077	1122113 (95%)	G/G
S01_17a	SAMN05725910	Vienna-8 Strain El Pino SIT Guatemala	1310581	1245388 (95%)	T/T
S01_1	SAMN05725911	Vienna-8 Strain El Pino SIT Guatemala	904644	846079 (93%)	T/T
S01_3	SAMN05725912	Panama Chiriqui Province Boquete Bajo Mono	1210362	1115886 (92%)	G/G
S01_5	SAMN05725913	USA HI Oahu Island	1537738	1440518 (93%)	G/G
S01_8	SAMN05725914	USA CA Camarillo Ventura County[a]	2339704	2207090 (94%)	G/G
S01_9	SAMN05725915	USA CA Camarillo Ventura County[a]	1809760	1707308 (94%)	G/G

[a] Collected from active SIT release areas

Individual ID	BioSample	Origin	Total	Retained	Assay AHMSY8D Genotype
S02_16b	SAMN05725916	Vienna-8 Strain El Pino SIT Guatemala	1609358	1515321 (94%)	G/T
S02_17b	SAMN05725917	Vienna-8 Strain El Pino SIT Guatemala	1333829	1267602 (95%)	T/T
S02_18	SAMN05725918	Guatemala San Juan Atoenango Finca Capetillo[a]	1719678	1575265 (91%)	G/G
S02_26	SAMN05725919	Panama Chiriqui Province Boquete Bajo Mono	1295507	1241994 (95%)	G/G
S02_34	SAMN05725920	USA HI Oahu Island	1600787	1481665 (92%)	G/G
S03_16c	SAMN05725921	Vienna-8 Strain El Pino SIT Guatemala	691838	658353 (95%)	G/T
S03_17c	SAMN05725922	Vienna-8 Strain El Pino SIT Guatemala	905631	845154 (93%)	T/T
S03_19	SAMN05725923	Guatemala San Juan Atoenango Finca Capetillo[a]	1285515	1196690 (93%)	G/G
S03_27	SAMN05725924	Panama Chiriqui Province Boquete Bajo Mono	1243597	1164547 (93%)	G/G
S03_35	SAMN05725925	USA HI Oahu Island	2432042	2280836 (93%)	G/G
S03_S12_88	SAMN05725926	Spain CastellÃşn Plana Alta[a]	2645570	2492651 (94%)	G/G
S03_S12_89	SAMN05725927	Spain CastellÃşn Plana Alta[a]	3535606	3311643 (93%)	G/G
S03_S12_90	SAMN05725928	Spain CastellÃşn Plana Alta[a]	2182977	2059037 (94%)	G/T
S03_S12_91	SAMN05725929	Spain CastellÃşn Plana Alta[a]	1845870	1704786 (92%)	G/T
S03_S12_92	SAMN05725930	Spain CastellÃşn Plana Alta[a]	1716711	1546522 (90%)	G/G
S03_S12_93	SAMN05725931	Spain CastellÃşn Plana Alta[a]	1177570	1102251 (93%)	G/T
S03_S12_94	SAMN05725932	Spain CastellÃşn Plana Alta[a]	1415587	1314503 (92%)	G/T
S03_S12_95	SAMN05725933	Spain CastellÃşn Plana Alta[a]	1547558	1446943 (93%)	G/G
S03_S12_96	SAMN05725934	Spain CastellÃşn Plana Alta[a]	2791660	2620379 (93%)	G/G
S04_16d	SAMN05725935	Vienna-8 Strain El Pino SIT Guatemala	1064900	1008470 (94%)	G/T
S04_17d	SAMN05725936	Vienna-8 Strain El Pino SIT Guatemala	920009	877561 (95%)	T/T
S04_28	SAMN05725937	Panama Chiriqui Province Boquete Bajo Mono	1122796	1059461 (94%)	G/G
S04_36	SAMN05725938	USA HI Oahu Island	2225668	2107069 (94%)	G/G
S04_S08_16	SAMN05725939	Morocco Marrakesh	5673870	4899547 (86%)	G/G
S04_S08_18	SAMN05725940	Morocco Marrakesh	2159270	1936597 (89%)	G/G
S04_S08_19	SAMN05725941	Morocco Marrakesh	1656947	1540832 (92%)	G/G
S04_S08_20	SAMN05725942	Morocco Marrakesh	1400183	1313605 (93%)	G/G
S04_S08_21	SAMN05725943	Mozambique Maputo	2395717	2205916 (92%)	G/G
S04_S08_24	SAMN05725944	Mozambique Maputo	1443061	1358599 (94%)	G/G
S04_S08_25	SAMN05725945	Mozambique Maputo	2539989	2373925 (93%)	G/G
S04_S08_48	SAMN05725946	South Africa Varnam Farm	2081261	1920816 (92%)	G/G
S04_S08_49	SAMN05725947	South Africa Varnam Farm	1952944	1830515 (93%)	G/G
S04_S08_50	SAMN05725948	South Africa Varnam Farm	1977466	1849416 (93%)	G/G
S04_S08_51	SAMN05725949	South Africa Varnam Farm	2143045	2016014 (94%)	G/G
S04_S08_55	SAMN05725950	Western Australia Perth West Swan[a]	1153403	981104 (85%)	G/G
S04_S08_56	SAMN05725951	Western Australia Perth West Swan[a]	1220248	1121684 (91%)	G/G
S04_S08_57	SAMN05725952	Western Australia Perth West Swan[a]	756636	700537 (92%)	G/G
S05_16e	SAMN05725953	Vienna-8 Strain El Pino SIT Guatemala	867810	814053 (93%)	G/T
S05_21	SAMN05725954	Guatemala San Juan Atoenango Finca Capetillo[a]	1748217	1648367 (94%)	G/G
S05_29	SAMN05725955	Panama Chiriqui Province Boquete Bajo Mono	1209910	1140517 (94%)	G/G
S06_44	SAMN05725956	Vienna-8 Strain El Pino SIT Guatemala	643736	616976 (95%)	G/T

[a] Collected from active SIT release areas

Individual ID	BioSample	Origin	Total	Retained	Assay AHMSY8D Genotype
S08_S17_100	SAMN05725957	Spain Valencia Camp de Morvedre[a]	3399629	3161497 (92%)	G/T
S08_S17_101	SAMN05725958	Spain Valencia Camp de Morvedre[a]	598491	559703 (93%)	G/T
S08_S17_102	SAMN05725959	Spain Valencia Camp de Morvedre[a]	1437473	1347375 (93%)	G/G
S08_S17_103	SAMN05725960	Spain Valencia Camp de Morvedre[a]	1495852	1406815 (94%)	G/G
S08_S17_104	SAMN05725961	Spain Valencia Camp de Morvedre[a]	463999	438049 (94%)	G/T
S08_S17_105	SAMN05725962	Spain Valencia Camp de Morvedre[a]	1443954	1359625 (94%)	G/T
S08_S17_106	SAMN05725963	Spain Valencia Camp de Morvedre[a]	1492664	1386793 (92%)	G/G
S08_S17_107	SAMN05725964	Spain Valencia Camp de Morvedre[a]	1543074	1435666 (93%)	G/T
S08_S17_98	SAMN05725965	Spain Valencia Camp de Morvedre[a]	2507092	2363446 (94%)	G/T
S08_S17_99	SAMN05725966	Spain Valencia Camp de Morvedre[a]	1601361	1432663 (89%)	G/T
S09_S13_27	SAMN05725967	Mozambique Mitucue Catholic Mission	2532145	2356698 (93%)	G/G
S09_S13_28	SAMN05725968	Mozambique Mitucue Catholic Mission	2287622	1944097 (84%)	G/G
S09_S13_29	SAMN05725969	Mozambique Mitucue Catholic Mission	1500432	1322501 (88%)	G/G
S09_S13_30	SAMN05725970	Mozambique Mitucue Catholic Mission	1065541	998281 (93%)	G/G
S09_S13_38	SAMN05725971	South Africa Bonnievale	1689731	1578944 (93%)	G/G
S09_S13_39	SAMN05725972	South Africa Bonnievale	3967839	3689950 (92%)	G/G
S09_S13_40	SAMN05725973	South Africa Bonnievale	1795073	1671892 (93%)	G/G
S09_S13_41	SAMN05725974	South Africa Bonnievale	1568257	1482197 (94%)	G/G
S09_S13_42	SAMN05725975	South Africa Bonnievale	2167389	2034380 (93%)	G/G
S09_S13_43	SAMN05725976	South Africa Riebeek Kasteel	2280718	2131968 (93%)	G/G
S09_S13_44	SAMN05725977	South Africa Riebeek Kasteel	2691196	2473694 (91%)	G/G
S09_S13_45	SAMN05725978	South Africa Riebeek Kasteel	2625435	2448871 (93%)	G/G
S09_S13_47	SAMN05725980	South Africa Riebeek Kasteel	2487272	2335632 (93%)	G/G
S16_S25_58	SAMN05725981	Madeira Island North Coast[a]	613758	582100 (94%)	G/T
S16_S25_59	SAMN05725982	Madeira Island North Coast[a]	966889	913652 (94%)	G/T
S16_S25_60	SAMN05725983	Madeira Island North Coast[a]	1831821	1730940 (94%)	G/G
S16_S25_61	SAMN05725984	Madeira Island North Coast[a]	2074564	1946363 (93%)	G/G
S16_S25_62	SAMN05725985	Madeira Island North Coast[a]	939030	894776 (95%)	G/G
S16_S25_63	SAMN05725986	Madeira Island North Coast[a]	996063	919352 (92%)	G/T
S16_S25_64	SAMN05725987	Madeira Island North Coast[a]	1494814	1392312 (93%)	G/G
S16_S25_65	SAMN05725988	Madeira Island North Coast[a]	1096931	1027014 (93%)	G/T
S16_S25_66	SAMN05725989	Madeira Island North Coast[a]	541380	505834 (93%)	G/G
S16_S25_67	SAMN05725990	Madeira Island North Coast[a]	671363	624838 (93%)	G/T
S27_2	SAMN05725991	Honduras Trujillo Colon	2580459	2387159 (92%)	G/G
S28_22	SAMN05725992	Honduras Trujillo Colon	1037512	972860 (93%)	G/G
S29_23	SAMN05725993	Honduras Trujillo Colon	1329212	1263267 (95%)	G/G
S29_S30_31	SAMN05725994	Reunion Island Ligne Paradis	2098510	1957428 (93%)	G/G
S29_S30_32	SAMN05725995	Reunion Island Ligne Paradis	2152001	2019919 (93%)	G/G
S30_24	SAMN05725996	Honduras Trujillo Colon	2132509	1997408 (93%)	G/G
S31_25	SAMN05725997	Honduras Trujillo Colon	3382497	3151705 (93%)	G/G
S04_S08_33	SAMN05726002	Senegal Keur Moussa	2360349	2228904 (94%)	G/G

[a] Collected from active SIT release areas

Individual ID	BioSample	Origin	Total	Retained	Assay AHMSY8D Genotype
S04_S08_34	SAMN05726003	Senegal Keur Moussa	4500413	4221251 (93%)	G/G
S04_S08_35	SAMN05726004	Senegal Keur Moussa	4229508	3950606 (93%)	G/G
S04_S08_36	SAMN05726005	Senegal Keur Moussa	2021251	1703378 (84%)	G/G
S04_S08_37	SAMN05726006	Senegal Keur Moussa	1948599	1817412 (93%)	G/G
8_3_2_1F	SAMN05727687	Mapping population	2941650	2732872 (92%)	G/T
8_3_2_1M	SAMN05727688	Mapping population	2913257	2728331 (93%)	G/T
8_3_2_2F	SAMN05727689	Mapping population	2500136	2028246 (81%)	G/T
8_3_2_2M	SAMN05727690	Mapping population	3047766	1869400 (61%)	G/T
8_3_2_5F	SAMN05727691	Mapping population	2036686	1644757 (80%)	G/T
8_3_2_5M	SAMN05727692	Mapping population	2894450	2361683 (81%)	G/T
8_3_2F	SAMN05727693	Mapping population	2672412	2488526 (93%)	T/T
8_3_2M	SAMN05727694	HiMed	2858875	2628710 (91%)	G/G
8_3F	SAMN05727695	Mapping population	4837377	4484323 (92%)	G/T
8_3M	SAMN05727696	Mapping population	3371578	3118376 (92%)	G/T
B8_3_2_1_10M	SAMN05727697	Mapping population	1365733	1083551 (79%)	T/T
B8_3_2_1_11M	SAMN05727698	Mapping population	2034085	1627945 (80%)	G/T
B8_3_2_1_12M	SAMN05727699	Mapping population	1790807	1518892 (84%)	G/T
B8_3_2_1_13M	SAMN05727700	Mapping population	1405218	1139920 (81%)	G/G
B8_3_2_1_14M	SAMN05727701	Mapping population	2205941	1787339 (81%)	G/T
B8_3_2_1_15M	SAMN05727702	Mapping population	2671415	2140710 (80%)	G/G
B8_3_2_1_16M	SAMN05727703	Mapping population	3260614	2424495 (74%)	G/G
B8_3_2_1_18M	SAMN05727704	Mapping population	2402772	1882473 (78%)	G/T
B8_3_2_1_20M	SAMN05727705	Mapping population	1602117	1323837 (82%)	G/T
B8_3_2_1_21F	SAMN05727706	Mapping population	256926	87285 (33%)	No Call
B8_3_2_1_21M	SAMN05727707	Mapping population	1889671	1540837 (81%)	G/G
B8_3_2_1_23M	SAMN05727708	Mapping population	2415767	1982851 (82%)	G/T
B8_3_2_1_24M	SAMN05727709	Mapping population	1982826	1668960 (84%)	G/T
B8_3_2_1_26M	SAMN05727710	Mapping population	2187415	1821334 (83%)	G/T
B8_3_2_1_2F	SAMN05727711	Mapping population	6495095	4799064 (73%)	G/G
B8_3_2_1_30F	SAMN05727712	Mapping population	381472	313076 (82%)	G/G
B8_3_2_1_7M	SAMN05727713	Mapping population	2894240	2358772 (81%)	G/T
B8_3_2_1_8M	SAMN05727714	Mapping population	2296871	1768730 (77%)	G/G
B8_3_2_1_9M	SAMN05727715	Mapping population	2703425	2118762 (78%)	G/G
B8_3_2_2_10M	SAMN05727716	Mapping population	2474885	2038947 (82%)	G/T
B8_3_2_2_17M	SAMN05727717	Mapping population	2529962	2146993 (84%)	G/T
B8_3_2_2_18M	SAMN05727718	Mapping population	1884957	1581003 (83%)	G/T
B8_3_2_2_19M	SAMN05727719	Mapping population	1843719	1594770 (86%)	G/T
B8_3_2_2_1F	SAMN05727720	Mapping population	462594	350306 (75%)	No Call
B8_3_2_2_20F	SAMN05727721	Mapping population	1912156	1620416 (84%)	G/G
B8_3_2_2_25F	SAMN05727722	Mapping population	5208936	4052703 (77%)	G/T
B8_3_2_2_29M	SAMN05727723	Mapping population	2233081	1742756 (78%)	G/T

Individual ID	BioSample	Origin	Total	Retained	Assay AHMSY8D Genotype
B8_3_2_2_33M	SAMN05727724	Mapping population	2184584	1764560 (80%)	G/G
B8_3_2_2_7M	SAMN05727725	Mapping population	3180504	2251814 (70%)	No Call
B8_3_2_5_10F	SAMN05727726	Mapping population	3293317	3071228 (93%)	G/T
B8_3_2_5_10M	SAMN05727727	Mapping population	3043529	2473708 (81%)	G/T
B8_3_2_5_11F	SAMN05727728	Mapping population	857033	806077 (94%)	G/G
B8_3_2_5_11M	SAMN05727729	Mapping population	4307523	3478879 (80%)	G/G
B8_3_2_5_16M	SAMN05727730	Mapping population	2240143	2084099 (93%)	G/T
B8_3_2_5_18M	SAMN05727731	Mapping population	1919964	1781499 (92%)	G/T
B8_3_2_5_19F	SAMN05727732	Mapping population	2381024	2245349 (94%)	G/T
B8_3_2_5_1F	SAMN05727733	Mapping population	2658815	2457947 (92%)	G/T
B8_3_2_5_1M	SAMN05727734	Mapping population	2959998	2444722 (82%)	G/T
B8_3_2_5_20M	SAMN05727735	Mapping population	7970101	6816124 (85%)	G/G
B8_3_2_5_21M	SAMN05727736	Mapping population	3809804	3400647 (89%)	G/T
B8_3_2_5_2F	SAMN05727737	Mapping population	1213307	1138933 (93%)	G/G
B8_3_2_5_2M	SAMN05727738	Mapping population	2455246	2025460 (82%)	G/G
B8_3_2_5_36F	SAMN05727739	Mapping population	3494981	3257786 (93%)	G/T
B8_3_2_5_39F	SAMN05727740	Mapping population	19686	7166 (36%)	No Call
B8_3_2_5_3M	SAMN05727741	Mapping population	3273164	2724779 (83%)	G/G
B8_3_2_5_44F	SAMN05727742	Mapping population	6018679	5169118 (85%)	No Call
B8_3_2_5_47F	SAMN05727743	Mapping population	2490682	2223007 (89%)	G/T
B8_3_2_5_48F	SAMN05727744	Mapping population	3047079	2820764 (92%)	G/T
B8_3_2_5_4M	SAMN05727745	Mapping population	3764060	3139155 (83%)	G/T
B8_3_2_5_5M	SAMN05727746	Mapping population	3810053	2959813 (77%)	G/T
B8_3_2_5_6M	SAMN05727747	Mapping population	3588630	2929143 (81%)	G/T
B8_3_2_5_7F	SAMN05727748	Mapping population	4353600	4033701 (92%)	G/T
B8_3_2_5_7M	SAMN05727749	Mapping population	2073687	1764352 (85%)	G/T
B8_3_2_5_8M	SAMN05727750	Mapping population	1639621	1281683 (78%)	G/G
B8_3_2_5_9M	SAMN05727751	Mapping population	3549326	2942248 (82%)	G/G
W8_3_2_1_10M	SAMN05727752	Mapping population	3633292	2755439 (75%)	T/T
W8_3_2_1_11M	SAMN05727753	Mapping population	4262999	3353459 (78%)	T/T
W8_3_2_1_12M	SAMN05727754	Mapping population	4322232	3230052 (74%)	T/T
W8_3_2_1_13M	SAMN05727755	Mapping population	5521555	4389289 (79%)	No Call
W8_3_2_1_14M	SAMN05727756	Mapping population	3632687	2841977 (78%)	T/T
W8_3_2_1_15M	SAMN05727757	Mapping population	2582761	2072238 (80%)	T/T
W8_3_2_1_16M	SAMN05727758	Mapping population	4090905	3166399 (77%)	T/T
W8_3_2_1_1M	SAMN05727759	Mapping population	4288480	2565157 (59%)	T/T
W8_3_2_1_2M	SAMN05727760	Mapping population	3948054	2961934 (75%)	T/T
W8_3_2_1_3M	SAMN05727761	Mapping population	2707350	2164414 (79%)	T/T
W8_3_2_1_4M	SAMN05727762	Mapping population	2041677	1638856 (80%)	T/T
W8_3_2_1_5M	SAMN05727763	Mapping population	3033032	2319803 (76%)	T/T
W8_3_2_1_6M	SAMN05727764	Mapping population	3027864	2395716 (79%)	T/T

Individual ID	BioSample	Origin	Total	Retained	Assay AHMSY8D Genotype
W8_3_2_1_7M	SAMN05727765	Mapping population	2840572	2271641 (79%)	T/T
W8_3_2_1_8M	SAMN05727766	Mapping population	3548919	2832074 (79%)	T/T
W8_3_2_1_9M	SAMN05727767	Mapping population	3922526	2752409 (70%)	T/T
W8_3_2_2_1F	SAMN05727768	Mapping population	2304322	1841230 (79%)	T/T
W8_3_2_2_2F	SAMN05727769	Mapping population	1305260	1100085 (84%)	No Call
W8_3_2_2_3F	SAMN05727770	Mapping population	1003775	846307 (84%)	T/T
W8_3_2_2_4F	SAMN05727771	Mapping population	2383444	1962463 (82%)	T/T
W8_3_2_2_5F	SAMN05727772	Mapping population	2947244	2247423 (76%)	T/T
W8_3_2_2_6F	SAMN05727773	Mapping population	1867467	1446740 (77%)	T/T
W8_3_2_2_6M	SAMN05727774	Mapping population	2408257	1997786 (82%)	T/T
W8_3_2_2_7F	SAMN05727775	Mapping population	1716867	1463539 (85%)	T/T
W8_3_2_2_8F	SAMN05727776	Mapping population	2359744	1890746 (80%)	T/T
W8_3_2_2_9F	SAMN05727777	Mapping population	1319579	1062245 (80%)	T/T
W8_3_2_5_10F	SAMN05727778	Mapping population	2508510	1975705 (78%)	T/T
W8_3_2_5_11F	SAMN05727779	Mapping population	2410671	1933448 (80%)	T/T
W8_3_2_5_11M	SAMN05727780	Mapping population	3033822	2421430 (79%)	No Call
W8_3_2_5_12M	SAMN05727781	Mapping population	1254414	986027 (78%)	T/T
W8_3_2_5_13M	SAMN05727782	Mapping population	2770878	2038340 (73%)	T/T
W8_3_2_5_15M	SAMN05727783	Mapping population	3189742	2494739 (78%)	T/T
W8_3_2_5_16M	SAMN05727784	Mapping population	2979475	2408543 (80%)	T/T
W8_3_2_5_17M	SAMN05727785	Mapping population	3920538	3134875 (79%)	T/T
W8_3_2_5_1F	SAMN05727786	Mapping population	2309898	1927067 (83%)	T/T
W8_3_2_5_1M	SAMN05727787	Mapping population	1609145	1272560 (79%)	T/T
W8_3_2_5_2F	SAMN05727788	Mapping population	2454374	2063575 (84%)	T/T
W8_3_2_5_2M	SAMN05727789	Mapping population	4832781	3755998 (77%)	T/T
W8_3_2_5_3F	SAMN05727790	Mapping population	2177120	1780385 (81%)	T/T
W8_3_2_5_3M	SAMN05727791	Mapping population	4150087	3295750 (79%)	T/T
W8_3_2_5_4F	SAMN05727792	Mapping population	1900085	1616996 (85%)	T/T
W8_3_2_5_4M	SAMN05727793	Mapping population	5627371	4138542 (73%)	T/T
W8_3_2_5_5F	SAMN05727794	Mapping population	1622242	1142554 (70%)	T/T
W8_3_2_5_5M	SAMN05727795	Mapping population	5639616	4123686 (73%)	T/T
W8_3_2_5_6F	SAMN05727796	Mapping population	2166229	1711359 (79%)	T/T
W8_3_2_5_6M	SAMN05727797	Mapping population	4479477	3254093 (72%)	T/T
W8_3_2_5_7F	SAMN05727798	Mapping population	2168339	1762066 (81%)	T/T
W8_3_2_5_7M	SAMN05727799	Mapping population	4913498	3766600 (76%)	T/T
W8_3_2_5_8F	SAMN05727800	Mapping population	1229450	989863 (80%)	T/T
W8_3_2_5_8M	SAMN05727801	Mapping population	4304956	3447530 (80%)	T/T
W8_3_2_5_9M	SAMN05727802	Mapping population	3849008	3053719 (79%)	T/T
