# Supplementary material for: A New Diagnostic Resource for Ceratitis capitata Strain Identification Based on QTL Mapping
Source: G3 (Bethesda). 2017 Sep 9;7(11):3637–47. doi: 10.1534/g3.117.300169 (PMC5677166; doi:10.1534/g3.117.300169)
Supplement: Supplementary file 4 [file 3637FileS1.pdf]

Table S1

GBS read counts per individual. Individual origin, number of raw reads, reads removed due to lack of restriction site, reads removed due to low quality, and number and proportion of reads retained per individual in GBS sequencing library.

Figure S1

Linkage map of *C. capitata* calculated using recombination in three mapping populations. Three related mapping populations were used to identify linkage groups. The linkage groups 4a, 4b, 5, 6a, 6b, 6c, and 6d contained scaffolds which could be anchored to chromosomes 4, 5, and 6 in *C. capitata*. Linkage groups A, B, C, and D do not contain scaffolds which can be anchored to a chromosome.

Figure S2

Allelic discrimination plot for wild-captures from Spain which cluster with mass-rearing Vienna strain flies based on genome-wide SNP genotypes and known control individuals. Six wild-captures from Spain that clustered with mass-rearing Vienna strain individuals based on DAPC are also identified as Vienna strain males based on the SNP assay.
